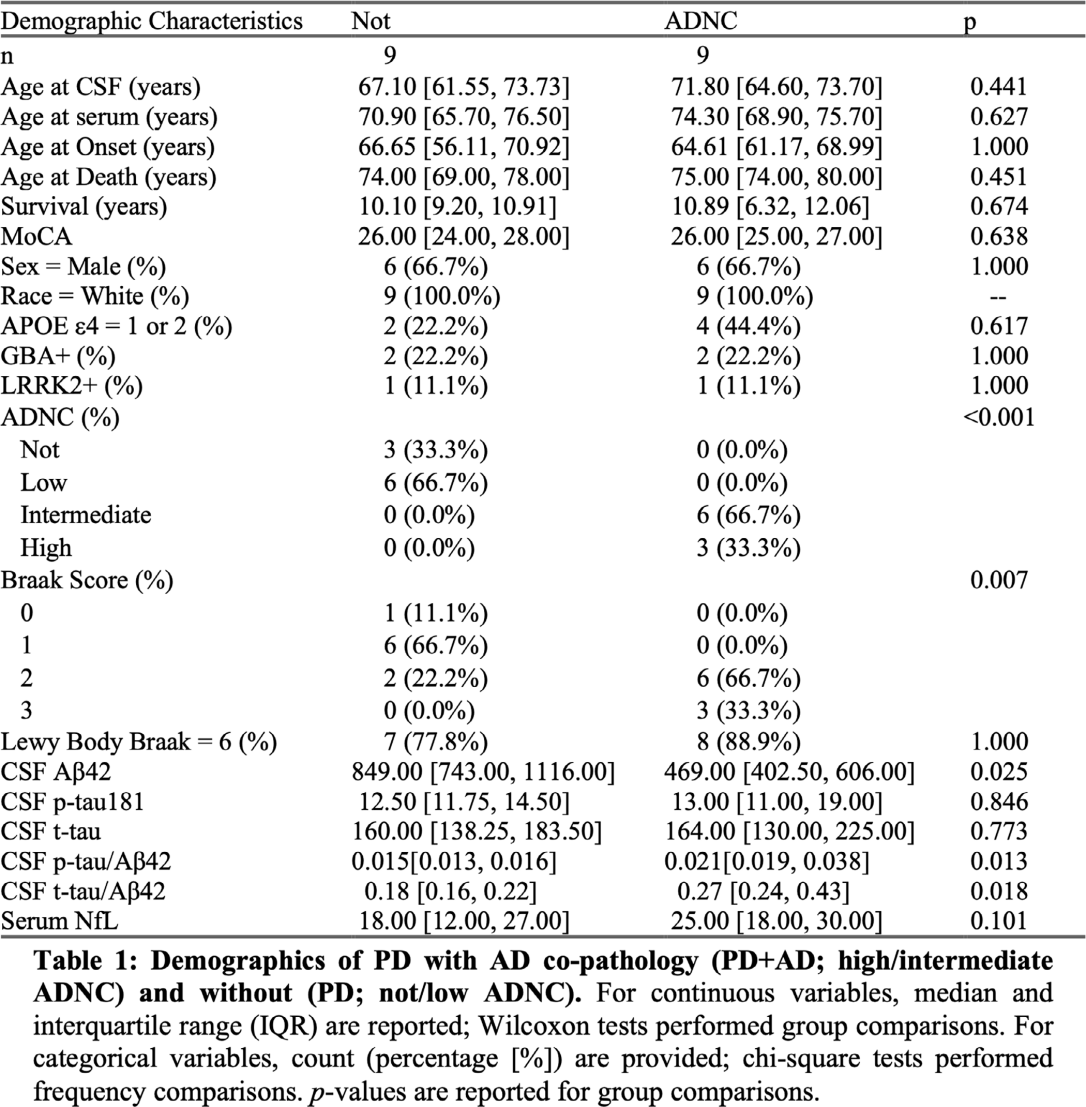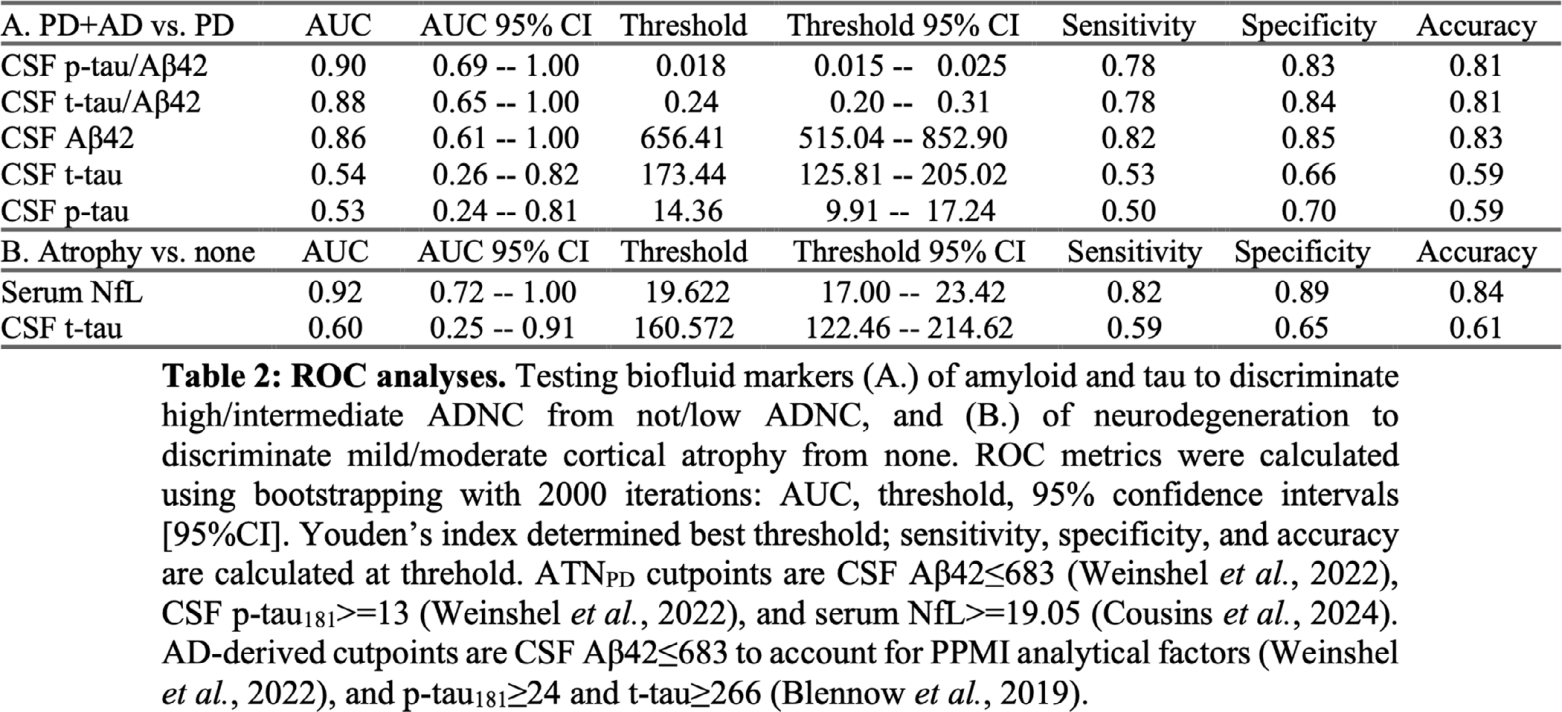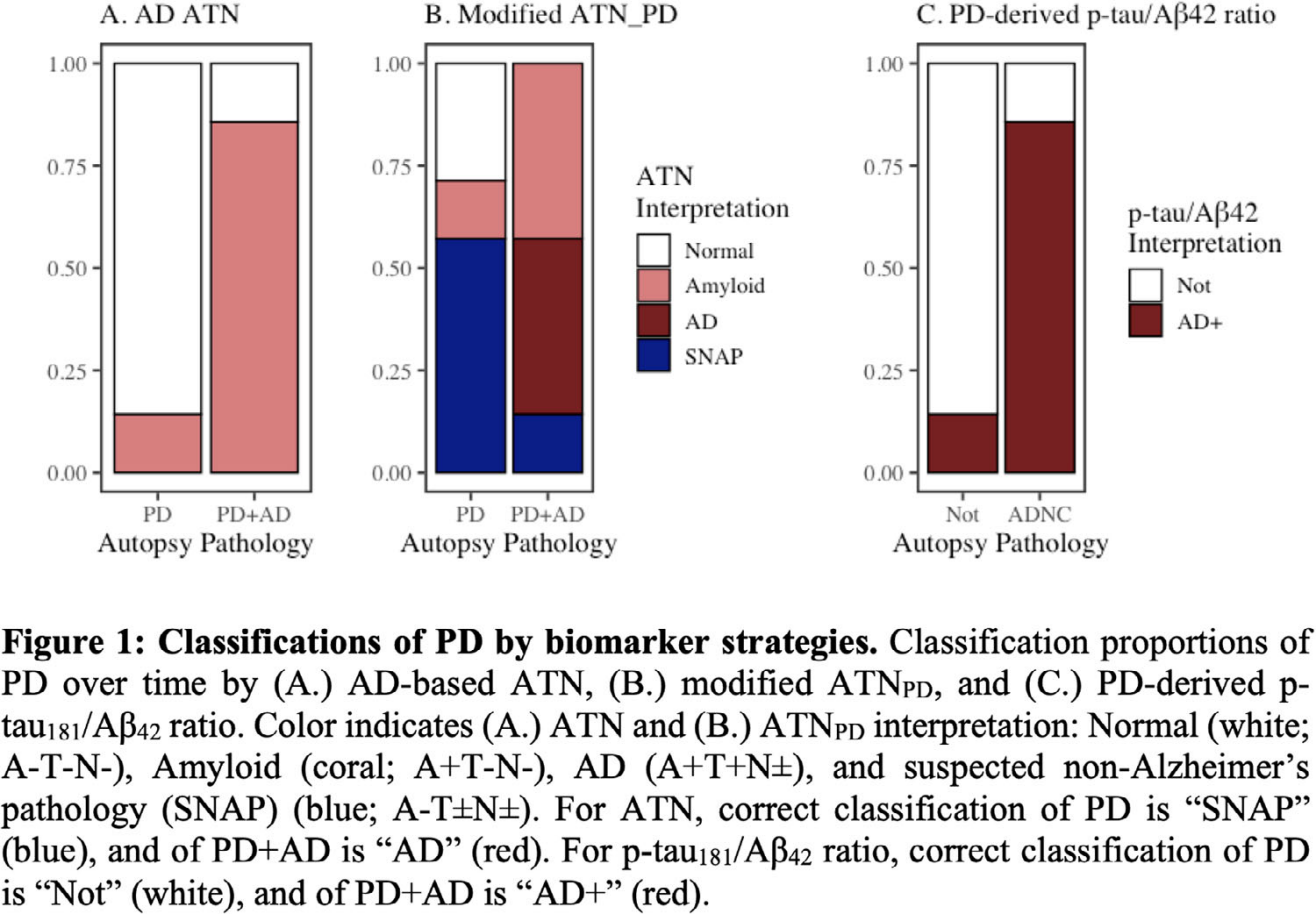# ATN_PD_ to improve detection of concomitant Alzheimer’s pathology in autopsy‐confirmed Parkinson’s disease

**DOI:** 10.1002/alz.091273

**Published:** 2025-01-09

**Authors:** Katheryn A Q Cousins, Ece Bayram, Douglas R. Galasko, Kristy S Hwang, Leslie M. Shaw, David J Irwin, David G Coughlin

**Affiliations:** ^1^ Department of Neurology, University of Pennsylvania, Philadelphia, PA USA; ^2^ University of California San Diego Parkinson and Other Movement Disorders Center, Department of Neurosciences, La Jolla, CA USA; ^3^ Department of Neurosciences, University of California San Diego, La Jolla, CA USA; ^4^ Section of Neurology, Long Beach Veterans Administration Healthcare System, Long Beach, CA USA; ^5^ Department of Pathology and Laboratory Medicine, Perelman School of Medicine, University of Pennsylvania, Philadelphia, PA USA

## Abstract

**Background:**

In Parkinson’s disease (PD), concomitant Alzheimer’s disease (AD) pathologic change (ADNC) is common and results in altered motor and cognitive phenotypes. However, detection of PD with AD (PD+AD) using biofluid markers is challenging. While decreased cerebrospinal fluid (CSF) β‐amyloid 1‐42 (Aβ_42_) strongly reflects β‐amyloid burden, PD subjects typically harbor lower CSF phosphorylated tau 181 (p‐tau_181_) and total tau (t‐tau) levels than healthy controls, which complicates detection of tau tangles and neurodegeneration. We previously tested PD‐specific application of the β‐amyloid/tau/neurodegeneration framework (ATN_PD_); combining CSF Aβ_42_, CSF p‐tau_181_, and serum neurofilament light (NfL) in a living PD cohort. ATN_PD_, using a lower CSF p‐tau_181_ cutpoint, predicted cognitive decline. However, ATN_PD_ cutpoints still must be validated against autopsy assessments of ADNC as gold‐standard. Here, we compare biomarker strategies in all available autopsy‐confirmed PD from the Parkinson’s Progression Markers Initiative (PPMI).

**Methods:**

Eighteen PD participants with autopsy‐confirmed Lewy body disease and antemortem biofluid were available for analysis (Table 1). PD+AD included high/intermediate ADNC (n=9); PD without AD (PD; n=9) included not/low ADNC. Cerebral cortical atrophy determined neurodegeneration (mild/moderate vs. none). CSF was assayed for Aβ_42_ (n=14), p‐tau_181_ (n=17), and t‐tau (n=17) using Roche cobas e 601; p‐tau_181_/Aβ_42_ and t‐tau_181_/Aβ_42_ ratios were calculated. Serum NfL was assayed using Simoa Quanterix (n=18). Biofluid measurements closest to autopsy were selected. Receiver operating characteristic (ROC) analyses with bootstrapping tested discrimination of PD+AD from PD using CSF biomarkers, and of neurodegeneration from not using CSF t‐tau and serum NfL.

**Results:**

ROC cutpoints for CSF Aβ_42_, p‐tau_181_, and serum NfL were equivalent to ATN_PD_ cutpoints, while p‐tau_181_ and t‐tau were lower than published AD‐cutpoints (Table 2). CSF p‐tau_181_/Aβ_42_, t‐tau_181_/Aβ_42_, Aβ_42_ and serum NfL had high area under the curve (AUC>0.80; Table 2A,2B). In contrast, CSF p‐tau_181_ and t‐tau demonstrated poor discrimination (Table 2A) and no difference between groups (Table 1), potentially due in part to low sample size. A chi‐square test confirmed classification is improved using ATN_PD_ and AD‐cutpoints (χ^2^=14, p=0.0015; Figure 1).

**Conclusions:**

PD‐specific biomarker strategies/cutpoints are needed to maximize detection of concomitant ADNC, but must be validated in larger autopsy cohorts.